# Unusual Presentation of Pelizaeus-Merzbacher Disease: Female Patient with Deletion of the Proteolipid Protein 1 Gene

**DOI:** 10.1155/2015/453105

**Published:** 2015-02-18

**Authors:** Teva Brender, Donna Wallerstein, John Sum, Robert Wallerstein

**Affiliations:** Silicon Valley Genetics Center, Santa Clara Valley Medical Center, San Jose, CA 95128, USA

## Abstract

Pelizaeus-Merzbacher disease (PMD) is neurodegenerative leukodystrophy caused by dysfunction of the proteolipid protein 1 (*PLP1*) gene on Xq22, which codes for an essential myelin protein. As an X-linked condition, PMD primarily affects males; however there have been a small number of affected females reported in the medical literature with a variety of different mutations in this gene. No affected females to date have a deletion like our patient. In addition to this, our patient has skewed X chromosome inactivation which adds to her presentation as her unaffected mother also carries the mutation.

## 1. Introduction

Pelizaeus-Merzbacher disease (PMD) is a rare neurodegenerative condition caused by hypomyelination of the central nervous system classified as a leukodystrophy. It is characterized by deterioration of coordination, motor abilities, and intellectual function. PMD is an X-linked condition due to mutations in the proteolipid protein 1 (*PLP1*) gene on Xq22, which codes for an essential myelin protein. Most reported individuals have duplications of or point mutations within this gene. As an X-linked condition, this primarily affects males. To date, there have been a small number of affected females reported in the medical literature; none of them are due to a deletion of this gene.

## 2. Case Report

**Figure 1:**
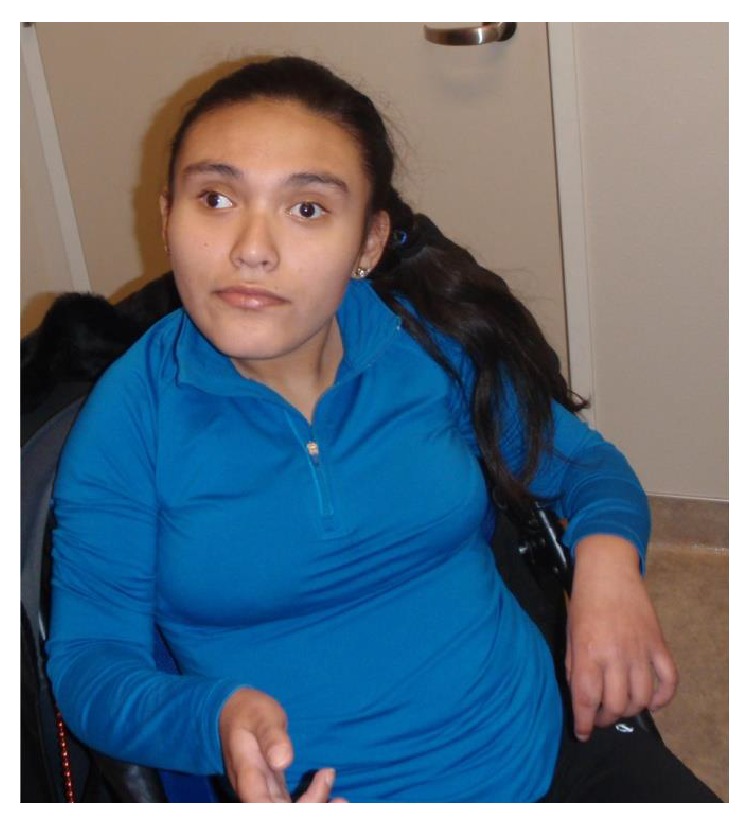
Photograph of the patient at the age of 16.

**Figure 2:**
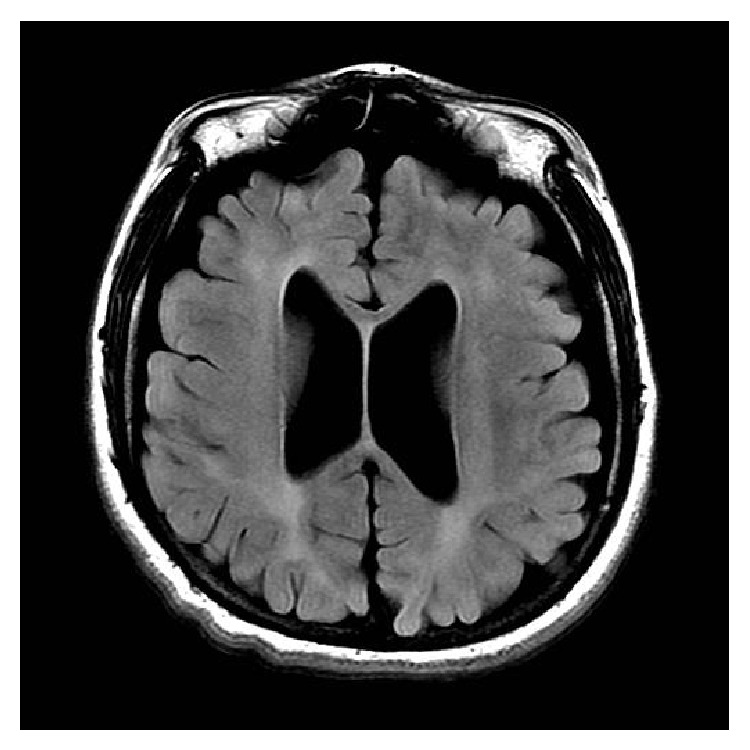
Axial MRI view of the brain at the age of 17 showing bilateral diffuse symmetric increased FLAIR signal in the subcortical and periventricular white matter with diffuse atrophic changes involving the supratentorial brain. Axial FLAIR image performed on a 3T MR GE scanner with TR: 9002, TE: 124.7.

**Figure 3:**
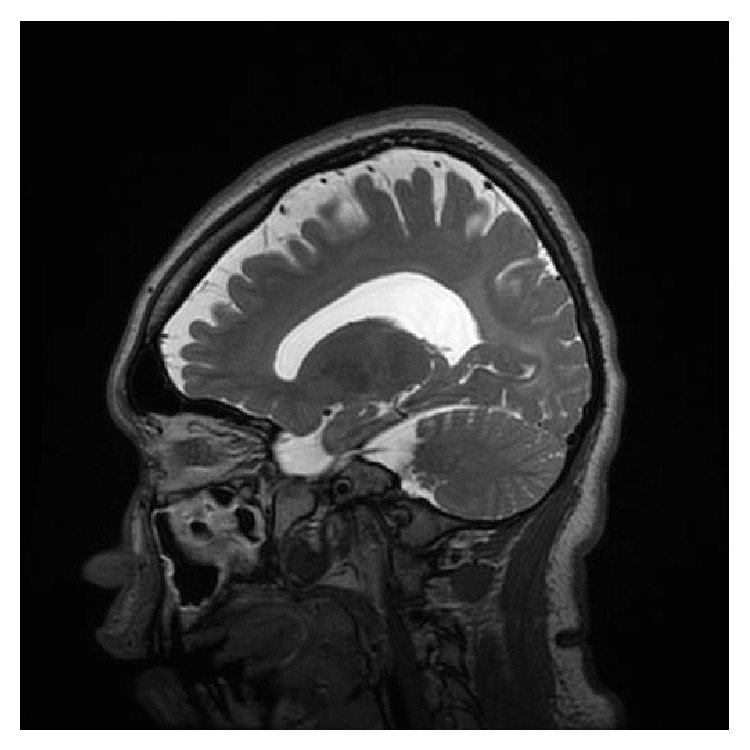
Sagittal MRI view of the brain at the age of 17 showing diffuse increased T2 signal in the subcortical and periventricular white matter demonstrating diffuse leukodystrophy. GE sagittal T2 CUBE image performed on a 3T MR GE scanner with TR: 3000, TE: 69.1.

This patient was a female born as the first child of her nonconsanguineous parents at 34 weeks of gestation after a pregnancy complicated by severe preeclampsia. At birth, she was noted to have nystagmus and at 6 months of age her parents were concerned with her delayed motor development due to spasticity that continued to progress. She developed limited ambulation with support after the age of 3 years after intensive physical therapy. Meaningful language never developed. At the age of 12, she was able to learn 5 signs that she was able to use for 2 years, but this skill was lost around the age of 14. Her family reports that they believe that she has receptive language in the presence of complete absence of expressive language. She continued to have nystagmus as well as exotropia. She developed episodes of agitation with biting and poorly controlled self-destructive behavior that was treated with carbamazepine. She developed progressive left leg weakness and stopped walking independently at the age of 7. MRI of the brain performed at ages of 4, 9, 13, and 17 showed linear increased T2 signal from the frontal horn to the occipital horn of the lateral ventricles bilaterally that did not change, consistent with a chronic dysmyelinating disease (see Figures 2 and 3). At the age of 16, her spasticity had improved due to intense physical therapy and administration of botulinum toxin (see Figure 1). A new onset seizure disorder began at the age of 16. EEG demonstrated abundant bursts of generalized spike, polyspike, and slow wave activity. At the age of 17, she was having daily seizures of short duration that are not treated with medication. She walked with support but could not bear weight. She could eat whole food with assistance and remained nonverbal. She has not experienced significant change for the last 3 to 4 years.

Genetic testing performed by chromosome microarray using the Affymetrix Cytoscan HD platform with 743,000 SNP probes showed a 712KB interstitial deletion on the X chromosome at position Xq22.2. There are 6 genes within this region including* NGFRAP1*,* TCEAL1*,* MORFL2*,* PLP1*,* RAB9B*, and* H2BFWT*. None of these have clinically reported phenotype except PLP1. For confirmation, direct testing of the* PLP1* gene for dosage (deletions and/or duplications) was performed by multiplex ligation dependent amplification (MLPA). This showed deletion of the* PLP1* gene of exons 1 through 7, which encompasses the entire gene.

X chromosome inactivation studies were performed via methylation-sensitive restriction digest followed by PCR and fragment analysis. This showed a nonrandom skewed X chromosome inactivation pattern of 81 : 19 by methylation analysis of the androgen gene locus. Ratios of 50 : 50 to 79 : 21 are considered random, ratios of 80 : 20 to 89 : 11 are moderately skewed, and ratios of 90 : 10 to 100 : 0 are highly skewed. This result together with the phenotype suggests that the X chromosome with the duplication is preferentially activated.

## 3. Discussion

Mutations or alterations of the* PLP1* gene cause hypomyelination and PMD. PMD is an X-linked disorder and as such typically only males are affected. Heterozygous females may be affected in one of two ways. When the* PLP1* mutation causes little or no oligodendrocyte apoptosis, the disorder acts like an X-linked dominant condition with variable penetrance as heterozygous females may develop neurodegenerative symptoms later in life. Conversely, heterozygous females may be affected by an X-linked recessive pattern through skewed X inactivation [1]. Because our patient developed symptoms associated with PMD in infancy, her condition is likely due to X-linked inheritance with skewed X inactivation, which was confirmed by studies as noted above. The genesis of this disorder then depends on 2 independent events of the gene deletion and skewed X chromosome inactivation.

After a review of the literature, we located 7 other cases of affected females with a genetic diagnosis of PMD in each case finding a mutation or duplication of the* PLP1* gene [2–4]. Our patient was the first affected female with a gene deletion.

Current understanding of* PLP1* gene mutations holds that deletions lead to a milder form of PMD [5]. However, our patient presents a fairly severe but stable phenotype and as such suggests that PMD patients deletions have a wider range of phenotypic expression. The 5 other genes in the deleted region may contribute to the severity of the phenotype. The fact that the causative mutation is a deletion may be related to the long period of stability the patient has experienced.

## 4. Conclusions

This case highlights the following.

1. Females can be affected, albeit rarely, with PMD so it needs to be considered in patients both males and females with progressing spasticity.
2. There is a wide spectrum of phenotypic expression of PMD.
3. The genetic mechanisms of this disorder are complex due to abnormalities in the* PLP1* gene of point mutations, gene duplications, and deletions. Deletions of the* PLP1* gene are very rare but are documented to cause PMD.
4. In addition to the* PLP1* gene abnormality, a second event of skewed X chromosome inactivation is needed for the disorder to manifest in a female.
